# Targeting MITF in the tolerance-phase

**DOI:** 10.18632/oncotarget.9423

**Published:** 2016-05-18

**Authors:** Imanol Arozarena, Michael P. Smith, Claudia Wellbrock

**Affiliations:** Manchester Cancer Research Centre, Wellcome Trust Centre for Cell-Matrix Research, The University of Manchester, Manchester, UK

**Keywords:** melanoma, BRAF, MITF, HIV-protease, therapy resistance

6 years ago melanoma was an untreatable cancer without an effective therapy beyond surgical excision, but today immune checkpoint- and MAP-kinase (MAPK)- pathway targeting therapies have significantly improved patient survival. Nevertheless, the plethora of reports on the inevitable development of resistance in the majority of patients treated with BRAF and MEK inhibitors has greatly distracted from the remarkable initial responses these drugs produce in patients with *BRAF* mutant melanoma.

In an attempt to better understand the onset of resistance to MAPK-targeting inhibitors in melanoma patients, we have focused on the initial response phase, when tumours shrink because melanoma cell survival is strictly dependent on MAPK-signalling. We identified an early cell autonomous-driven non-mutational ‘tolerance phase’, in which melanoma cells in order to adapt to the loss of the vital MAPK signals re-wire their signalling and enhance expression of the MIcrophthalmia Transcription Factor (MITF), one of the major drivers of melanoma cell identity [1]. In line with our previous findings [2], we could demonstrate that by up-regulating MITF expression melanoma cells can withstand the MAPKinhibitor induced toxicity even before the establishment of mutational acquired resistance. This suggested that targeting MITF during the ‘tolerance phase’ could sensitize melanoma cells to MAPK-pathway inhibitors, thereby providing a window of opportunity for therapeutic intervention that would allow extending the efficacy of these drugs. Indeed, the HIV-protease inhibitor nelfinavir, which we identified as a potent down-regulator of MITF expression, profoundly increased the cytotoxicity of MAPK-inhibitors and reduced the development of resistance [1].

Although these pre-clinical results are encouraging, there are several questions that require further exploration. One of the potential concerns suggested in light of our findings is the possibility that MITF down-regulation could turn melanoma cells into so-called ‘MITF^low^ cells’, melanoma cell sub-populations found in ~20% of melanomas that are no longer driven by MITF but rather display a gene expression signature governed by receptor tyrosine kinase (such as AXL, EGFR, ERBB3), WNT5A or NFkB signalling [3–5]. Importantly, these MITF^low^/ AXL^high^ cells are greatly resistant to MAPK-inhibitors [3–5], and ~50% of melanomas identified in patients relapsed on MAPK-inhibitor treatment display reduced MITF expression possibly linked to the AXL^high^-signature. While this suggest a selection for reduced MITF expression in half the melanomas that progress on MAPK-inhibitor treatment alone, it is not known whether this frequency would change in patients that would escape a nelfinavir/MAPK-inhibitor combination therapy, where during treatment, the MITF down-regulated cells are in fact the most vulnerable cells.

Another concern arising from targeting MITF is the possibility of ‘creating’ a population with ‘pro-metastatic’ properties. MITF regulates many cellular functions in melanoma cells, including differentiation, proliferation and survival [6]. Many groups including ours have observed that reduced levels of MITF trigger increased invasive behaviour, and accordingly MITF^low^ cells have been assigned a so called ‘invasive signature’ [7]. While this has greatly contributed to the idea that only MITF^low^ cells have metastatic potential, it is yet to be proven whether low levels of MITF confer an advantage in every step of the metastatic cascade. There are indeed gaps in our knowledge, and for instances the role of MITF in the survival of circulating melanoma cells or in promoting intra- or extravasation as well as growth in distant melanoma metastases, has not been fully addressed.

The above-mentioned concerns clearly highlight the need to characterize the biology of cells that have acquired resistance to MAPK-inhibitor/nelfinavir combination therapy. What are the key signalling nodes driving their survival and proliferation? What is the invasive and metastatic potential of cells that escape combinatorial therapies? Is MITF down-regulation maintained after Nelfinavir treatment cessation and if so what are the consequences? To answer these questions will be crucial in order to decide whether a MAPK-inhibitor/nelfinavir combination therapy can be safely moved into clinical trials.

Another important finding from our study is that a MEK-inhibitor/nelfinavir combination is also effective in *NRAS* mutant melanoma cells [1]. While this finding in itself is exciting as the options for targeting *NRAS* mutant melanoma are very limited, it also suggests that most mechanisms of BRAF inhibitor resistance leading to pathway reactivation upstream of MEK could be targeted by the MEK-inhibitor/nelfinavir combination. Indeed, we show that a pre-clinical model for *NRAS*/*BRAF*/*PTEN* mutant melanoma can be effectively treated with a MEK-inhibitor/nelfinavir combination [1]. Following on from this, with the advent of ERK inhibitors another therapeutic opportunity might offer itself to test nelfinavir combinations with the hope to further prolong the clinical effects of MAPK pathway inhibitors. Thus, while there are still several open questions that need to be addressed, the findings in Smith et al clearly send a positive message as they demonstrate that there is still room for improvement in therapies using MAPK-pathway targeting drugs in melanoma.

As a final note, the crucial observation that in the majority of melanomas on treatment with MAPKinhibitors MITF is significantly increased might lead to other therapeutic benefits, as the MITF induced upregulation of melanoma differentiation antigens could impact on the immune-microenvironment and lead to an improvement of the responses to immunotherapy approaches. Overall the Smith et al study makes a strong case for the use of MITF as predictive marker and suggests that monitoring MITF expression levels throughout treatment could help improving therapy responses.
